# Behavioral Paradigms and Methodological Variability in Aluminum Chloride-Induced Rat Models of Alzheimer’s Disease: A Structured Review

**DOI:** 10.3390/biology15090690

**Published:** 2026-04-28

**Authors:** Adrian-Florentin Dragomir, Aurelian Zugravu, Smaranda Stoleru, Elena Poenaru, Maria Carina Dumitrescu, George Albu, Teodora-Nicola Tomescu, Gabriela Raluca Ivan, Maria Georgiana Lacatus, Aurelia Cristiana Barbu, Silvia Fratea, Oana Andreia Coman, Ion Fulga

**Affiliations:** 1 Faculty of Medicine, “Carol Davila” University of Medicine and Pharmacy, 050474 Bucharest, Romania; adrian-florentin.dragomir@drd.umfcd.ro (A.-F.D.); aurelian.zugravu@umfcd.ro (A.Z.); maria-carina.dumitrescu@rez.umfcd.ro (M.C.D.); george.albu2023@stud.umfcd.ro (G.A.); teodora-nicola.tomescu2023@stud.umfcd.ro (T.-N.T.); gabriela-raluca.ivan2023@stud.umfcd.ro (G.R.I.); maria-georgiana.lacatus2023@stud.umfcd.ro (M.G.L.); aurelia-cristiana.barbu@drd.umfcd.ro (A.C.B.); oana.coman@umfcd.ro (O.A.C.); ion.fulga@umfcd.ro (I.F.); 2 Service Anesthesie-Reanimation, Groupe Hospitalier de la Pitié-Salpêtrière, CEDEX 13, 75651 Paris, France; silvia.fratea@aphp.fr

**Keywords:** Alzheimer’s disease, aluminum chloride, animal models, cognitive impairment, behavioral assessment, neurodegeneration, rat models

## Abstract

Aluminum chloride (AlCl_3_)-induced rat models are widely used to investigate Alzheimer-like neurodegeneration, yet substantial methodological variability limits cross-study comparability. A structured synthesis focused specifically on the methodological architecture of these models, including dose, exposure duration, route of administration, and behavioral assessment, remains lacking. This review aimed to synthesize the behavioral paradigms used to assess learning and memory in rat models of aluminum chloride-induced Alzheimer’s disease, with particular emphasis on dose, duration, and route of administration. A structured narrative review incorporating systematic elements was conducted following PRISMA-informed procedures using PubMed, Web of Science, and Scopus. The reviewed literature showed a predominance of oral administration, low-to-moderate AlCl_3_ doses and subchronic exposure durations, most commonly 31–60 days. Behavioral assessment was dominated by hippocampal-dependent paradigms, particularly the Morris water maze and Y-maze. Across studies, AlCl_3_ exposure was associated with multidomain behavioral impairment accompanied by consistent hippocampal and cortical histopathological abnormalities and convergent biochemical and molecular changes, including cholinergic dysfunction, oxidative stress, neuroinflammation, and amyloid- and tau-related alterations. Overall, the available literature does not support a standardized experimental protocol or a clear overall dose–effect or duration–effect relationship. Greater harmonization of study design is needed to improve reproducibility and translational relevance.

## Simple Summary

Alzheimer’s disease is a major cause of memory loss and mental decline, but its causes are still not fully understood and effective treatments remain limited. For this reason, researchers often use animal studies to explore how the disease develops and to test possible treatments. Among these, studies in rats exposed to aluminum chloride are widely used because they can reproduce several features of Alzheimer-like brain damage. However, these studies differ greatly in the amount of aluminum chloride given, how long it is administered, how it is delivered and which memory tests are used. This makes results difficult to compare and may reduce the value of these models for future research. In this review, we examined how these rat models are methodologically designed and how cognition is assessed. We found that most studies rely on only a few memory tests and that the experimental methods vary considerably. Better standardization could improve the reliability of results and support the development of more useful models for Alzheimer’s disease research.

## 1. Introduction

Alzheimer’s disease (AD) is a major neurodegenerative disorder with a substantial impact on quality of life and an increasing public health burden. Clinically, it is characterized by progressive cognitive decline, with variable involvement of multiple domains, including memory, language, executive function, and visuospatial abilities [1,2,3,4]. Neuropathologically, AD is characterized by the accumulation of beta-amyloid oligomers and the aggregation of tau protein, leading to the formation of neurofibrillary tangles and neuropil threads. These alterations are accompanied by chronic neuroinflammation, with accumulation of pro-inflammatory cytokines, reactive oxygen species, and other mediators that contribute to synaptic dysfunction, neuronal degeneration, and impaired neurogenesis [5,6,7].

In the absence of curative therapies or treatments that can fundamentally modify disease progression, biomedical research relies heavily on animal models to elucidate pathogenic mechanisms and evaluate novel therapeutic strategies [6,7,8,9]. At present, both genetic models and models induced by pharmacological or toxic agents are widely used. Although transgenic models are highly valuable, they are often costly and less accessible. Moreover, they typically reproduce only selected aspects of the disease and do not fully capture the complexity of sporadic AD, which accounts for the vast majority of clinical cases (over 95%) [8,9,10,11,12,13].

In recent years, chemically and pharmacologically induced animal models of AD have been used increasingly, driven by the need for more accessible and cost-effective systems that better reflect the multifactorial nature of sporadic AD and offer greater experimental flexibility. A wide range of agents have been used to induce Alzheimer-like phenotypes, including proteins and chemical compounds such as scopolamine, streptozotocin, aluminum chloride (AlCl_3_), amyloid-β peptides, and okadaic acid, as well as other substances capable of triggering neuroinflammatory and neurodegenerative processes. Aluminum, a neurotoxic metal known to affect cholinergic neurotransmission, is widely used in experimental studies of learning and memory in laboratory animals [2,14,15,16]. It can cross the blood–brain barrier and accumulate in vulnerable brain regions, such as the hippocampus and frontal cortex, where it induces histological, biochemical, and behavioral alterations that resemble key features of human Alzheimer’s pathology [14,15,16,17].

Aluminum is most commonly administered in the form of aluminum chloride [16,18]. AlCl_3_ can be delivered orally or intraperitoneally and, less frequently, by intracerebroventricular or intrahippocampal injection. The doses used in experimental studies span a broad range, from very low levels (e.g., 4.2 mg/kg body weight) to as high as 500 mg/kg body weight, and exposure may be acute, lasting only a few days, or chronic, extending over several months [15,16,18]. A variety of animal models have been used, including mice, rats, zebrafish, Drosophila melanogaster, and non-human primates, although rodent models remain the mainstay of preclinical research [19,20,21]. Compared with mice, rats are particularly well suited for behavioral and histological studies because of their more complex behavioral repertoire and larger brain size. In AlCl_3_-induced models, learning and memory are assessed using a range of behavioral paradigms targeting different cognitive domains: the Morris water maze (MWM) for spatial memory, the Y-maze for working memory, passive avoidance tasks for associative memory, and the novel object recognition (NOR) test for short- and long-term recognition memory [22,23,24,25,26,27].

Despite the large number of published studies, a structured synthesis of how AlCl_3_-induced rat models of Alzheimer-like disease are experimentally designed is still lacking. In particular, the literature remains highly heterogeneous with regard to AlCl_3_ dose, exposure duration, route of administration, and the behavioral paradigms used to assess learning and memory. Most existing reviews focus primarily on the molecular mechanisms of aluminum neurotoxicity, the histopathological changes induced by exposure, or the therapeutic effects of candidate interventions, rather than on the methodological features that define the behavioral model itself. As a result, it remains difficult for researchers to determine which experimental regimens are most commonly used and how behavioral assessment is typically performed across studies. This methodological variability has also made it difficult to establish a standardized experimental protocol for AlCl_3_ dose, exposure duration, and behavioral battery selection.

The aim of this review is to provide a structured overview of the main methodological characteristics of AlCl_3_-induced rat models used in AD research. Specifically, we sought to synthesize the AlCl_3_ doses, exposure durations, routes of administration, and learning and memory tests most frequently employed in these models, while also examining how these experimental variables shape the behavioral outcomes reported across studies.

## 2. Materials and Methods

This review was conducted as a structured narrative synthesis that incorporates systematic elements in order to enhance methodological rigor. Given the substantial heterogeneity across studies in terms of AlCl_3_ dose, exposure duration, route of administration, behavioral paradigms and outcome reporting, the findings were synthesized using a stratified descriptive approach rather than direct cross-study comparison of individual outcomes. A formal quantitative meta-analysis was not performed because the included studies differed substantially in experimental design, behavioral paradigms and the interpretation of outcomes relative to study-specific control groups.

### 2.1. Search Strategy and Data Sources

The search strategy was designed to identify original experimental studies investigating cognitive impairment induced by AlCl_3_ in rat models of AD, using at least one validated learning and/or memory test. Searches were performed in the following databases: PubMed, Web of Science Core Collection and Scopus. Search queries were conducted independently in each database, in accordance with their specific indexing structures and search requirements.

The main search terms included “rats,” “Alzheimer’s disease,” “learning and memory tests,” and “aluminium chloride”. No restrictions on year of publication were applied in order to capture the temporal evolution of research interest in this field and to include as many relevant studies as possible. Study screening was performed by four independent reviewers. The search process was carried out between 7 and 25 December 2025. All retrieved records were exported to Microsoft Excel, where duplicate entries were identified and removed prior to screening.

Disagreements between reviewers were resolved by discussion and consensus. The extracted variables included the year of publication, the dose of AlCl_3_, the duration of exposure, the route of administration, the cognitive tests used and the main reported cognitive outcomes.

### 2.2. Eligibility Criteria

The review was restricted to rat studies in order to preserve species-level consistency across behavioral paradigms and model induction protocols.

#### 2.2.1. Inclusion Criteria

Studies were considered eligible for inclusion if they fulfilled all of the following criteria:

- original experimental investigations conducted in rats;
- use of AlCl_3_ as the sole agent to induce an Alzheimer-type model in rats in at least one experimental group;
- inclusion of at least one validated learning and/or memory test, with reporting of objective cognitive outcomes based on quantifiable behavioral parameters;
- presence of an appropriate control group;
- full-text availability, written in English.

#### 2.2.2. Exclusion Criteria

Studies were excluded if they met any of the following conditions:

- conducted in species other than rats (e.g., mice, fish, insects, non-human primates) or in human subjects;
- reporting exclusively biochemical or molecular markers in the absence of cognitive assessment;
- employing combined or mixed models of cognitive impairment;
- failing to establish an experimental Alzheimer-type model;
- absence of a distinct experimental group in which AlCl_3_ was administered alone to induce the model;
- assessing only anxiety-like behavior, depressive-like behavior, or locomotor activity without the inclusion of cognitive tests;
- publication type limited to reviews, meta-analyses, editorials or conference abstracts;
- lack of full-text availability or publication in a language other than English.

### 2.3. Selection of Studies

**Figure 1:**
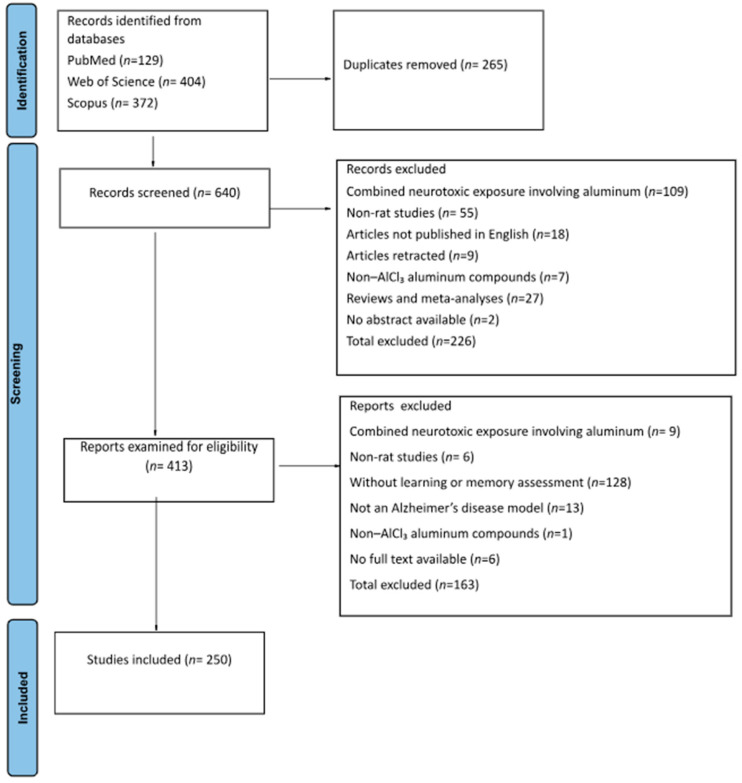
PRISMA flow diagram of study selection.

Study inclusion and exclusion were performed using Preferred Reporting Items for Systematic Reviews and Meta-Analyses (PRISMA)-informed procedures. Initially, all records were organized in an Excel file and the first step consisted of the removal of duplicate entries. Subsequently, a screening procedure based on titles and abstracts was applied, leading to the exclusion of a subset of records that did not meet the predefined eligibility criteria. The results of the selection process are presented in Figure 1.

## 3. Results

### 3.1. Temporal Distribution of Included Studies

**Figure 2:**
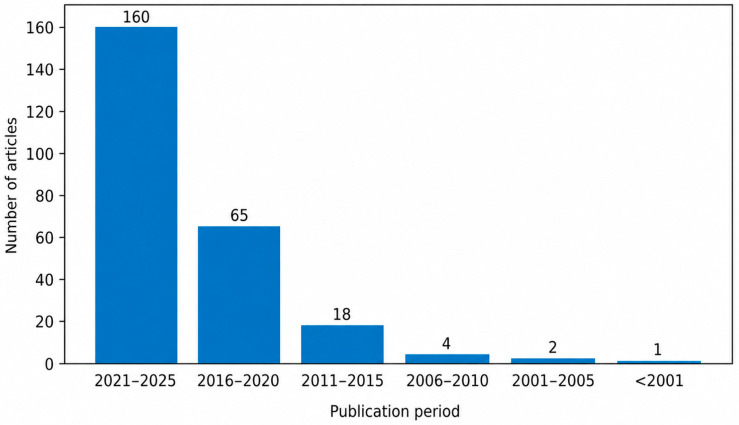
Temporal distribution of the included studies according to publication period. The figure shows a marked increase in publications after 2015, with a clear predominance of studies published between 2021 and 2025.

The included articles were grouped into five-year intervals (2021–2025, 2016–2020, 2011–2015, 2006–2010, 2001–2005, and before 2001) to illustrate the temporal evolution of research interest in this field. The number of studies identified in each interval is shown in Figure 2.

Out of the 250 included studies, 160 were published between 2021 and 2025 [16,28,29,30,31,32], followed by 65 published between 2016 and 2020 [33,34,35,36,37] and 18 published between 2011 and 2015 [38,39,40,41,42]. Earlier periods were represented by substantially fewer publications, with only four studies between 2006 and 2010 [43], two between 2001 and 2005 [44], and one study published prior to 2001 [45].

### 3.2. Aluminum Chloride Administration Routes in Experimental Rat Models of Alzheimer’s Disease

**Figure 3:**
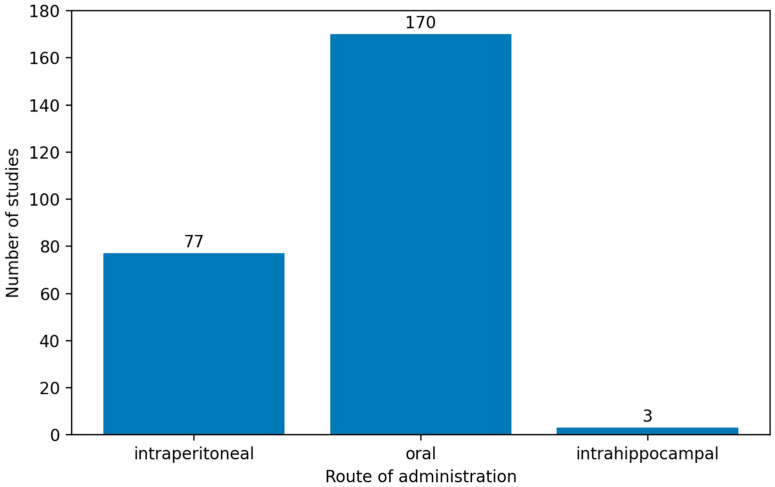
Distribution of the included studies according to the route of aluminum chloride administration (intraperitoneal, oral, and intrahippocampal). Oral administration predominated, whereas intrahippocampal administration was rarely used.

In experimental rat models of AD, AlCl_3_ has been administered via different routes, including intraperitoneal [46,47,48,49,50] and oral delivery [51,52,53,54,55], as well as direct intrahippocampal injection to induce localized neurodegeneration [56,57]. The distribution of the included studies according to the route of administration is presented in Figure 3.

Oral administration was the most frequently employed route, accounting for 170 of the 250 included studies, followed by intraperitoneal administration with 77 studies, whereas the intrahippocampal approach was rarely used, being identified in only 3 studies.

### 3.3. Aluminum Chloride Dosing Regimens Stratified by Route of Administration

Of the 250 included studies, 243 AlCl_3_ dose values reported in mg/kg body weight were eligible for descriptive quantitative analysis, comprising 77 intraperitoneal and 166 oral administration protocols. This number was lower than the total number of included studies because some articles reported more than one dosing regimen, while others expressed AlCl_3_ exposure in formats that could not be directly converted into mg/kg body weight (e.g., ppm in feed or drinking water, μg per animal, or intermittent fixed-dose schedules). These protocols were therefore considered only in the qualitative synthesis [58,59,60].

For ease of interpretation, AlCl_3_ doses were stratified into predefined intervals: ≤50, 51–100, 101–150, 151–200, 201–250, and >250 mg/kg body weight. This categorization provided a structured overview of the dose ranges most frequently employed across experimental rat models of AD, stratified by route of administration.

#### 3.3.1. Intraperitoneal Dosing Regimens

**Figure 4:**
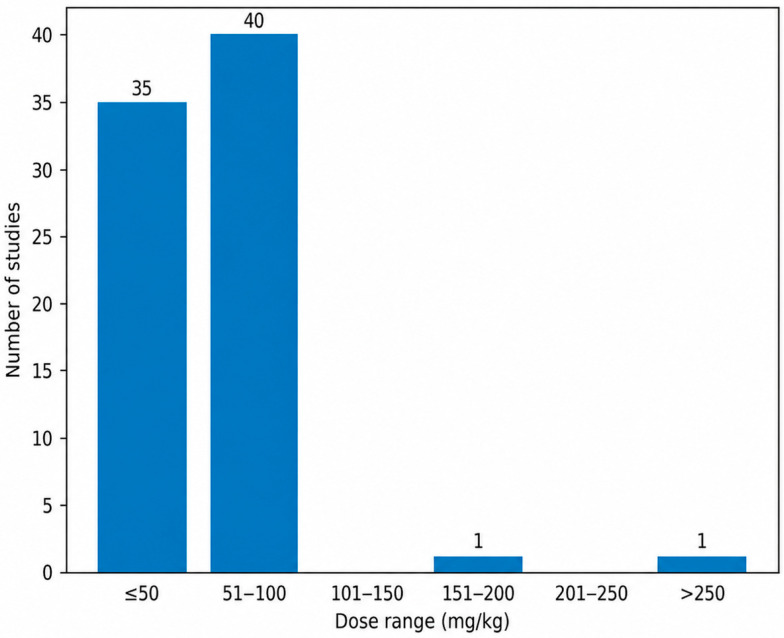
Intraperitoneal Aluminum Chloride Dose Ranges in Experimental Rat Models of Alzheimer’s Disease. Most intraperitoneal protocols were concentrated within the low-to-moderate dose ranges.

Intraperitoneal administration of AlCl_3_ was most frequently performed using doses ≤50 mg/kg and within the 51–100 mg/kg range [61,62,63,64,65,66,67,68,69]. Higher dose intervals were only sporadically represented, with a single study reporting doses in the 151–200 mg/kg range [70] and one study employing doses >250 mg/kg [71]. No intraperitoneal studies reported doses between 101–150 mg/kg or 201–250 mg/kg. As illustrated in Figure 4, this distribution highlights a pronounced clustering of intraperitoneal protocols within the low-to-moderate dose ranges.

**Figure 5:**
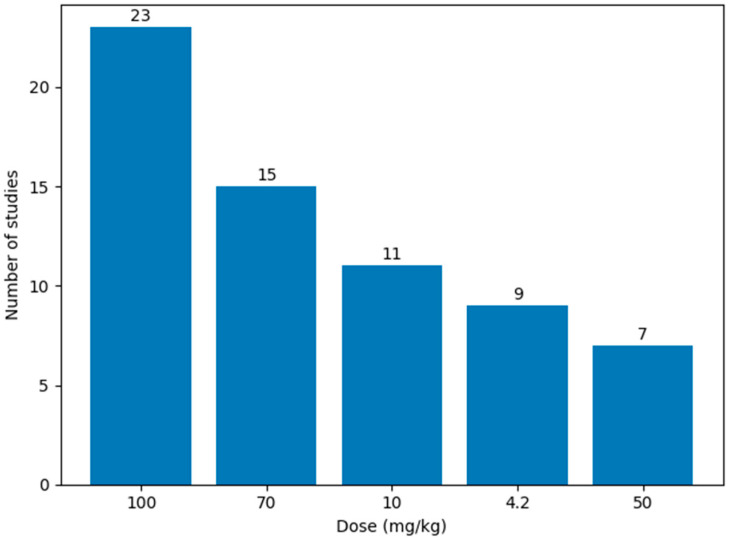
Top Five Aluminum Chloride Doses Used in Intraperitoneal Rat Models of Alzheimer’s Disease. The 100 mg/kg dose was the most frequently employed intraperitoneal regimen.

As shown in Figure 5, within intraperitoneal paradigms, the most frequently employed dose was 100 mg/kg [72,73,74,75], followed by 70 mg/kg [76,77,78,79] and 10 mg/kg [61,62,64]. Lower doses, such as 4.2 mg/kg [28,29] and 50 mg/kg [80,81,82,83], were also commonly used, whereas all other dose levels were reported only sporadically. Overall, intraperitoneal regimens ranged from a minimum of 4.2 mg/kg to a maximum of 300 mg/kg.

#### 3.3.2. Oral Dosing Regimens

**Figure 6:**
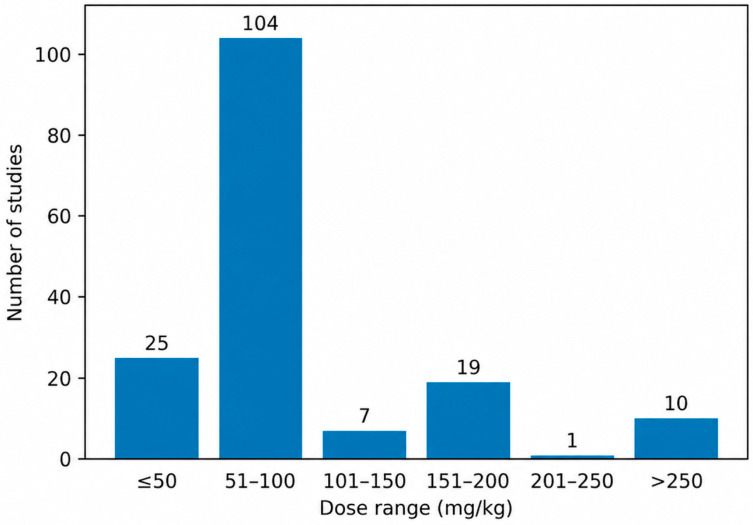
Oral Aluminum Chloride Dose Ranges in Experimental Rat Models of Alzheimer’s Disease. Oral protocols were predominantly clustered within the 51–100 mg/kg interval.

Oral administration of AlCl_3_ was most commonly performed within the 51–100 mg/kg range [84,85,86,87]. Lower doses (≤50 mg/kg) [88,89,90,91,92] were also frequently employed, whereas higher dose intervals were progressively less common. Doses between 101–150 mg/kg [93,94,95,96] and 151–200 mg/kg [97,98,99,100] were reported in a smaller number of studies, and few investigations employed doses exceeding 250 mg/kg [101,102,103]. The 201–250 mg/kg interval was represented by only one study [104]. Overall, this pattern indicates a strong concentration of oral protocols within the low-to-moderate dose ranges, with a marked preference for the 51–100 mg/kg interval, as shown in Figure 6.

**Figure 7:**
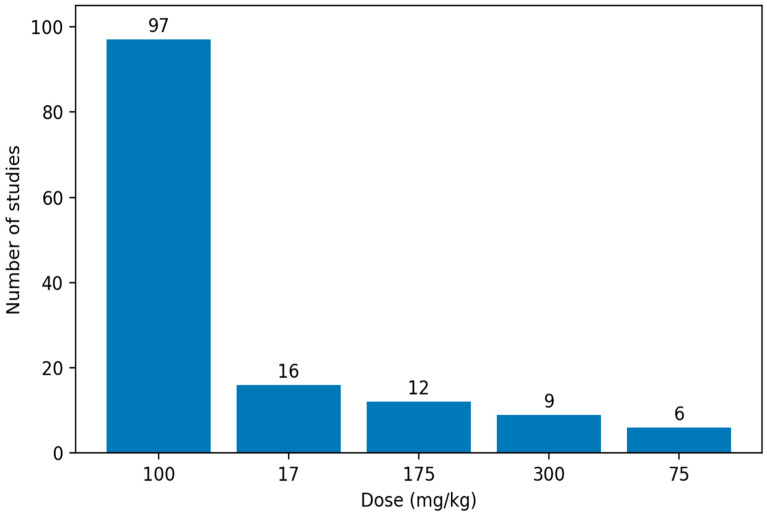
Top Five Aluminum Chloride Doses Used in Oral Rat Models of Alzheimer’s Disease. The 100 mg/kg dose was the most frequently employed oral regimen.

Within oral administration paradigms, a pronounced clustering at the individual dose level was observed. As shown in Figure 7, the most frequently employed dose was 100 mg/kg [105,106,107,108,109], followed by 17 mg/kg [110,111,112,113] and 175 mg/kg [114,115,116,117]. Higher doses, such as 300 mg/kg [118,119,120,121], as well as intermediate doses such as 75 mg/kg [122,123,124,125], were also repeatedly used, whereas all other dose levels were reported only sporadically. Overall, oral regimens ranged from a minimum of 10 mg/kg to a maximum of 500 mg/kg.

In experimental rat models of AD employing oral AlCl_3_ administration, several studies adopted alternative exposure strategies. These included sequential regimens combining an initial high-dose phase with prolonged exposure via drinking water, intermittent administration at fixed intervals (e.g., every other day), or continuous passive exposure through AlCl_3_-containing water provided ad libitum. In these paradigms, the actual amount of aluminum ingested cannot be precisely expressed in mg/kg, as it depends on individual drinking behavior and solution concentration [58,59,60].

#### 3.3.3. Intrahippocampal Dosing Regimens

Across intrahippocampal paradigms, AlCl_3_ was administered as a single, focal injection directly into the CA1 region of the hippocampus using stereotaxic techniques. Two studies employed an identical dose of 3.7 × 10^−4^ g/kg body weight, delivered unilaterally into the left hippocampus, thereby inducing a localized lesion in a structure critical for learning and memory. In contrast, a third study included multiple experimental groups receiving single intrahippocampal injections of AlCl_3_ at different doses (1–200 μg per rat) via a stereotaxically implanted guide cannula, enabling precise delivery into the CA1 region [56,57,59].

### 3.4. Aluminum Chloride Exposure Duration Stratified by Route of Administration

The assessment of exposure duration was performed on the same cohort of 243 studies that were included in the dose analysis. To facilitate interpretation and allow structured comparison across studies, exposure durations were grouped into four predefined intervals: 1–7 days, 8–30 days, 31–60 days, and >60 days.

#### 3.4.1. Intraperitoneal Exposure Duration

**Figure 8:**
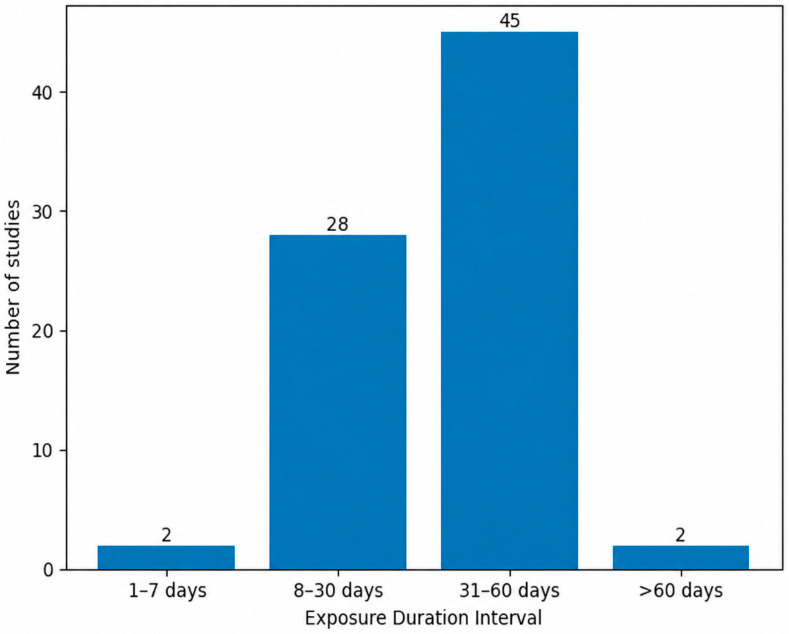
Intraperitoneal Aluminum Chloride Exposure Duration Intervals in Experimental Rat Models of Alzheimer’s Disease. Most studies used subchronic intraperitoneal exposure durations, particularly within the 31–60 day interval.

Among the 77 analyzed studies, only 2 employed very short exposure periods (1–7 days) [71,126], whereas the majority used intermediate durations (Figure 8). Specifically, 28 studies reported exposure periods between 8 and 30 days [127,128,129,130,131,132,133], and 45 studies used durations between 31 and 60 days [134,135,136,137,138,139], making this the most frequently applied interval. Long-term exposure exceeding 60 days was comparatively rare, being identified in only two studies [33,69]. This distribution highlights a clear predominance of subchronic AlCl_3_ administration protocols in experimental AD models. The shortest reported exposure consisted of a single day of AlCl_3_ administration, whereas the longest protocols extended beyond 60 days, reaching up to 70 days in some studies.

#### 3.4.2. Oral Exposure Duration

**Figure 9:**
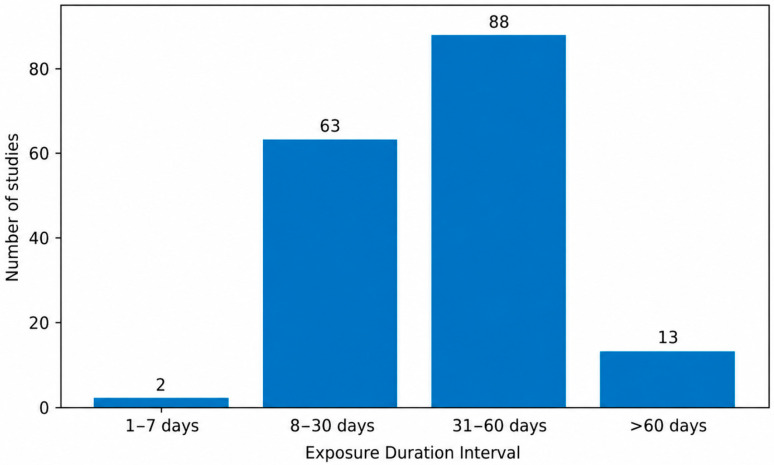
Oral Aluminum Chloride Exposure Duration Intervals in Experimental Rat Models of Alzheimer’s Disease. Oral studies were also concentrated within intermediate exposure durations, especially 31–60 days.

Oral AlCl_3_ exposure durations were predominantly concentrated within the intermediate time frames (Figure 9). Most studies employed treatment periods of 31–60 days [140,141,142,143,144,145,146,147,148,149], followed by protocols lasting 8–30 days [150,151,152,153,154,155,156,157,158,159,160]. Very short exposures (1–7 days) were rarely used [71,118], whereas prolonged regimens exceeding 60 days were uncommon [161,162,163,164,165,166,167,168]. The shortest reported oral exposure lasted 5 days, whereas the longest protocols extended to 180 days.

### 3.5. Behavioral Tests Used to Assess Learning and Memory in Experimental Rat Models of Alzheimer’s Disease

Behavioral learning and memory tests provide a quantitative assessment of cognitive impairment in experimental AD rat models, allowing an objective evaluation of induced cognitive deficits.

Different forms of learning and memory rely predominantly on specific cerebral structures. For example, the prefrontal cortex generally coordinates working memory and social learning, the frontal and parietal cortices are involved in executive functions, the hippocampus and entorhinal cortex mediate spatial learning and memory, the dorsal hippocampus supports incidental learning and the amygdala is central to emotional learning. For each of these functional domains, dedicated behavioral learning and memory tests have been developed to selectively probe the corresponding neural substrates.

In the analyzed articles, the principal learning and memory tests were grouped into seven categories: the NOR test, passive avoidance tests, the MWM, the Y-maze, the radial arm maze, the T-maze, and other less frequently used paradigms (e.g., the Barnes maze and adapted versions of the elevated plus maze).

**Figure 10:**
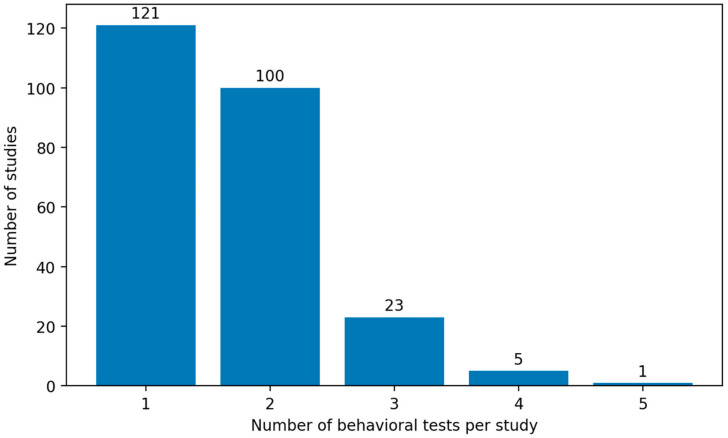
Number of Behavioral Learning and Memory Tests per Study in Experimental Rat Models of Alzheimer’s Disease. Most studies relied on one or two behavioral paradigms, whereas broader multimodal behavioral assessment was uncommon.

Of the 250 included studies, the majority employed a limited number of behavioral paradigms to assess cognitive function as shown in Figure 10. Most frequently, a single behavioral test was used (121 studies) [169,170,171,172,173,174,175,176,177,178], closely followed by protocols incorporating two tests (100 studies) [179,180,181,182,183,184,185,186,187,188]. In contrast, the use of more extensive testing batteries was uncommon: only 23 studies employed three behavioral assays [189,190,191,192,193,194,195,196,197,198], five studies included four tests [165], and a single study applied five distinct paradigms [32]. This distribution indicates that experimental models of AlCl_3_-induced cognitive impairment in rats typically rely on one or two behavioral measures to characterize learning and memory deficits, whereas comprehensive multimodal behavioral assessment remains relatively rare.

### 3.6. Behavioral Paradigms Used for the Assessment of Learning and Memory in Experimental Rat Models of Alzheimer’s Disease, Stratified by Route of Administration

**Figure 11:**
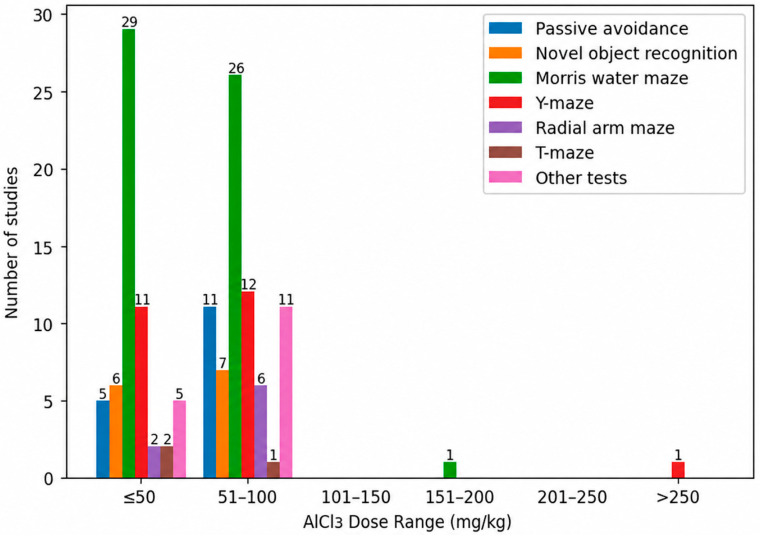
Learning and Memory Tests across Intraperitoneal Aluminum Chloride Dose Ranges in Experimental Rat Models of Alzheimer’s Disease. At low-to-moderate doses, behavioral assessment was dominated by the Morris water maze and Y-maze.

Figure 11 illustrates the distribution of behavioral paradigms used to assess learning and memory across different AlCl_3_ dose ranges in studies employing intraperitoneal administration. At the lower dose ranges (≤50 mg/kg and 51–100 mg/kg), the behavioral assessment was dominated by a core set of paradigms, with the MWM being the most frequently employed test [75,199,200] followed by the Y-maze [201], passive avoidance [202,203] and NOR test [182]. At doses between 151 and 200 mg/kg, cognitive performance was assessed exclusively using the T-Maze [70], whereas at doses exceeding 250 mg/kg, the only behavioral paradigm employed was the Y-maze test [71].

**Figure 12:**
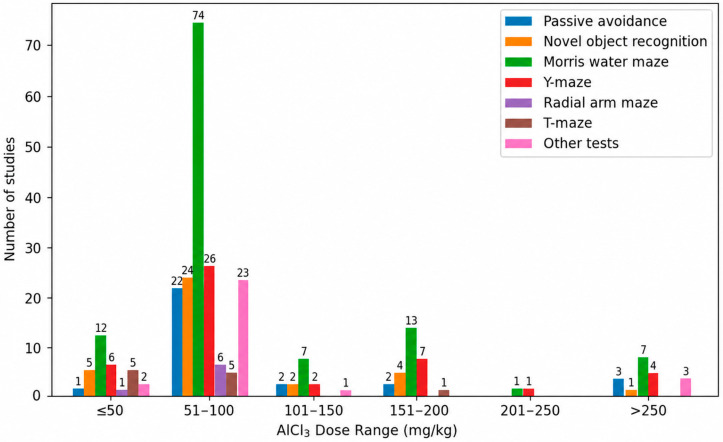
Learning and Memory Tests across Oral Aluminum Chloride Dose Ranges in Experimental Rat Models of Alzheimer’s Disease. The Morris water maze predominated across oral dose ranges, with more limited diversity of behavioral paradigms at higher doses.

Figure 12 illustrates the distribution of behavioral paradigms used to assess learning and memory across different AlCl_3_ dose ranges in studies employing oral administration. At the lower and intermediate dose ranges (≤50 mg/kg and 51–100 mg/kg), cognitive evaluation was dominated by a core set of paradigms, with the MWM being by far the most frequently employed test [204,205,206,207,208,209,210,211,212,213], followed by the Y-maze [214,215,216,217,218,219,220,221,222,223], NOR [224,225,226,227,228], and passive avoidance tasks [229,230,231,232,233,234,235,236,237,238,239].

At intermediate doses (101–150 mg/kg and 151–200 mg/kg), the same methodological pattern was preserved, with the MWM remaining the principal tool for cognitive assessment [240,241], complemented sporadically by Y-maze [242,243,244,245] and NOR paradigms. At higher dose ranges (201–250 mg/kg and >250 mg/kg), behavioral testing became more restricted, with only a limited number of paradigms being applied, predominantly the MWM and the Y-maze [104,246].

Across behavioral paradigms, AlCl_3_ exposure was associated with a broad pattern of behavioral impairment rather than with an isolated deficit restricted to a single task [65,135]. The most commonly affected domain was spatial learning and memory, most often assessed by the MWM, in which AlCl_3_-treated rats generally showed longer escape latencies during acquisition, poorer retrieval during probe trials, and reduced time spent in the target quadrant [65,85,199]. Working memory deficits were also frequently reported, particularly in Y-maze paradigms, where spontaneous alternation performance was reduced, and in radial arm maze protocols, where both working and reference memory errors increased [96,135]. Less frequently, T-maze paradigms were also used, and AlCl_3_-treated rats showed marked impairment of spontaneous alternation, consistent with deficits in spatial working memory and exploratory capacity [70]. Recognition memory was commonly impaired in NOR tasks, with treated animals showing reduced preference for the novel object and lower discrimination indices [66,155]. Associative or aversive memory deficits were likewise observed in passive avoidance and related retention-based paradigms, typically reflected by shorter step-through latencies or prolonged transfer latencies [39,135]. Some studies also used the modified elevated plus maze to assess learning and memory, most often through transfer latency–based measures [135]. In addition, several studies reported changes in exploratory behavior, anxiety-related performance, or motor function in tasks such as the open field, beam balance, and rotarod [39,66,96]. Overall, these findings indicate that the behavioral phenotype produced by AlCl_3_ is multidimensional, encompassing deficits in spatial, working, and recognition memory, together with broader alterations in exploratory behavior, anxiety-related performance, and motor function.

### 3.7. Histopathological Findings in Aluminum Chloride-Induced Rat Models of Alzheimer’s Disease

Histopathological abnormalities were reported in a substantial subset of the included studies and most consistently involved the hippocampus, particularly the CA1 and CA3 subfields and the dentate gyrus [39,65,66]. Across these studies, AlCl_3_ exposure was commonly associated with neuronal degeneration, disruption of normal cytoarchitecture, shrunken or pyknotic neurons, and vacuolative changes [39,70,87,88]. In some studies, hippocampal injury was further supported by Nissl staining, which showed reduced intact neuronal counts in the CA1, CA3, and dentate gyrus regions, while Congo red staining demonstrated β-amyloid plaque deposition in the CA1 region [65]. These findings support the view that the hippocampus is one of the principal anatomical targets of AlCl_3_ neurotoxicity in rat models of Alzheimer-like disease.

Beyond the hippocampus, several studies also described cortical and extra-hippocampal structural abnormalities [31,39,63,70,88]. Reported cortical changes included neuronal degeneration, vascular congestion or hemorrhagic changes, and glial alterations, while some reports also described perineuronal and perivascular edema [31,39,63,88]. Some studies additionally documented cerebellar involvement, including Purkinje cell degeneration and reduced granular layer density [247]. Amyloid-related deposition, increased Aβ immunoreactivity, and, in some cases, neurofibrillary tangle-like alterations were also reported, suggesting that at least a subset of AlCl_3_ rat models reproduces structural features associated with Alzheimer-like neurodegeneration [31,65,66,85,87]. Although imaging was reported far less frequently than histopathology, the limited available ultrastructural observations were broadly consistent with these structural findings [31,43].

### 3.8. Biochemical and Molecular Alterations in Aluminum Chloride-Induced Rat Models of Alzheimer’s Disease

Biochemical and molecular findings were broadly consistent with the behavioral and histopathological abnormalities described across the included studies. One of the most common patterns was evidence of oxidative imbalance, with AlCl_3_ exposure commonly associated with increased lipid peroxidation markers, including malondialdehyde (MDA) and thiobarbituric acid reactive substances (TBARS), together with reductions in endogenous antioxidant defenses such as superoxide dismutase (SOD), catalase (CAT), glutathione (GSH), glutathione peroxidase (GPx), and total antioxidant capacity (TAC) [31,36,117,135,205,207]. In parallel, cholinergic dysfunction was frequently reported, most often as increased acetylcholinesterase (AChE) activity. A small number of studies, however, described reduced acetylcholinesterase activity, possibly reflecting differences in model induction, sampled brain region, and assay methodology [31,36,39,43,52,63].

Inflammatory and neurotrophic alterations were also repeatedly identified. Across multiple studies, AlCl_3_ administration increased pro-inflammatory mediators such as tumor necrosis factor alpha (TNF-α), interleukin-1 beta (IL-1β), and interleukin-6 (IL-6), and in several reports this was accompanied by activation of broader inflammatory signaling pathways, including nuclear factor kappa B (NF-κB), inducible nitric oxide synthase (iNOS), cyclooxygenase-2 (COX-2), receptor for advanced glycation end products (RAGE), and microglial markers [31,66,117,161,207]. Some studies additionally reported vascular- or injury-related molecular changes, such as increased vascular cell adhesion molecule-1 (VCAM-1) and reduced vascular endothelial growth factor A (VEGF-A), while others described reductions in brain-derived neurotrophic factor (BDNF) and disturbances in monoaminergic neurotransmission, including decreases in dopamine and serotonin [88,205,209]. Together, these findings support the view that AlCl_3_ exposure induces a combined oxidative, inflammatory, and neurochemical disturbance rather than a single isolated molecular abnormality.

A substantial subset of studies further reported molecular alterations more closely aligned with Alzheimer-like pathology. These included increases in amyloid precursor protein (APP), amyloid beta (Aβ) or Aβ1–42, beta-site amyloid precursor protein-cleaving enzyme 1 (BACE1), β-secretase, and γ-secretase, as well as tau- or phosphorylated tau (pTau)-related abnormalities [36,66,135,136,161,204]. In several studies, these changes were accompanied by activation of apoptotic pathways, reflected by increased Bcl-2-associated X protein (Bax), cytosolic cytochrome c, and caspase-3, -8, or -9, together with reductions in anti-apoptotic markers such as B-cell lymphoma 2 (Bcl-2) and, in some reports, downregulation of Akt/GSK-3β signaling [31,72,86,135]. Beyond apoptosis, some studies also implicated necroptotic signaling, with increased hippocampal phosphorylation of receptor-interacting protein kinase 1 (RIP1), receptor-interacting protein kinase 3 (RIP3), and mixed lineage kinase domain-like protein (MLKL), increased cylindromatosis (CYLD) expression, and reduced cellular inhibitor of apoptosis 1 and 2 (cIAP1/2), supporting a possible contribution of regulated necrotic pathways to AlCl_3_-induced neurodegeneration [65]. Additional disease-relevant findings included increased α-synuclein, reduced nestin, altered cyclic guanosine monophosphate (cGMP) signaling, and changes in autophagy-related markers [86,88]. Overall, the biochemical and molecular data indicate that the AlCl_3_ model is characterized by convergent cholinergic, oxidative, inflammatory, amyloid-related, tau-related, pro-apoptotic, and necroptotic alterations, which provide a mechanistic substrate for the multidomain cognitive deficits observed across behavioral paradigms.

## 4. Discussion

The temporal distribution of publications reveals a marked and progressive increase in research interest in this field over the past two decades. The clear predominance of studies published between 2021 and 2025 suggests that this topic has gained substantial scientific attention only relatively recently. This trend likely reflects the growing global burden of AD, the continuous search for disease-modifying strategies and the increasing use of experimental models to explore novel therapeutic approaches. The sharp rise in publications after 2015 may also be linked to advances in molecular and behavioral neuroscience techniques, which have enabled more detailed investigation of neurodegenerative mechanisms [247,248,249,250,251,252,253,254,255,256].

The predominance of oral administration indicates a preference for exposure paradigms that approximate chronic environmental or dietary aluminum intake. The frequent use of the intraperitoneal route likely reflects its methodological reliability, enabling accurate dose control and reproducible systemic exposure. In contrast, intrahippocampal administration is rarely used, possibly because of its invasive nature [127,128,129,130,257,258,259,260,261,262,263,264,265].

The clustering of intraperitoneal AlCl_3_ doses within the ≤100 mg/kg range indicates a clear preference for low-to-moderate dosing paradigms that are sufficient to induce cognitive and neuropathological alterations while limiting excessive systemic toxicity. The repeated use of specific doses such as 100, 70, and 10 mg/kg suggests that these regimens have become empirically standardized in the field. Conversely, the limited use of very high doses likely reflects concerns regarding nonspecific toxic effects that extend beyond Alzheimer-like pathology [64,72,73,74,75,76,77,78,79].

A similar pattern is observed for oral AlCl_3_ administration, where the predominance of the 51–100 mg/kg interval indicates reliance mainly on low-to-moderate dosing. The clustering around 100 mg/kg implies that this dose has emerged as a practical reference point for model induction. Compared with intraperitoneal protocols, oral regimens display greater variability, including both lower and higher doses, possibly reflecting attempts to simulate heterogeneous patterns of chronic exposure. However, paradigms based on aluminum-containing drinking water or intermittent dosing schedules, while potentially more ecologically relevant, introduce variability in actual intake and reduce dose precision [266,267,268,269,270,271,272,273,274,275].

Regarding intracerebral administration of AlCl_3_**,** the reviewed studies targeted the hippocampal CA1 region, a structure critically involved in learning and memory and selectively vulnerable to neurotoxic insults. This focal stereotaxic approach enables the assessment of direct aluminum-induced hippocampal neurotoxicity while minimizing systemic effects [56,57,59].

Comparative analysis of exposure duration across administration routes shows a predominance of subchronic paradigms. Both oral and intraperitoneal studies predominantly employed exposure intervals between 31 and 60 days suggesting that this timeframe represents a balance between achieving measurable neurotoxic effects and avoiding excessive systemic toxicity [143,144,145,146,147,148,149].

The broader range of long-term protocols observed in oral studies, including exposures extending up to 180 days, further supports the notion that oral models aim to reproduce gradual accumulation and chronic neurotoxicity. While this is highly relevant to human exposure, it may also increase variability due to differences in gastrointestinal absorption, distribution and aluminum metabolism [161,162,163,164,165,166].

The predominance of studies employing only one or two behavioral paradigms suggests that cognitive assessment in AlCl_3_-induced models is often based on relatively narrow behavioral sampling. Although single-test approaches may detect clear behavioral impairments, they are unlikely to capture the multidimensional nature of cognition, which depends on coordinated activity across multiple neural systems [175,176,177,178,179,180,181,182,183,184].

In intraperitoneal models, behavioral assessment of learning and memory relies predominantly on a limited set of hippocampal-dependent paradigms, particularly the MWM and the Y-maze. This focus emphasizes spatial and recognition memory domains but may only partially reflect the broader cognitive disturbances characteristic of Alzheimer-like pathology. Expanding behavioral batteries to include multiple cognitive domains may provide a more comprehensive characterization of cognitive impairment in these models [199,200,201].

A similar pattern is observed in oral exposure models, where the MWM predominates across dose ranges, highlighting hippocampal-dependent spatial memory as the primary outcome. While the high sensitivity of this task to hippocampal dysfunction explains its popularity, recurrent reliance on a single paradigm constrains cognitive profiling [207,208,209,210,211,212,213,214,215,216].

The use of similar behavioral tests across dose ranges may provide some consistency in behavioral assessment, but it does not eliminate the broader methodological heterogeneity that limits direct cross-study comparison. In addition, the limited diversity of behavioral paradigms at higher doses may further constrain the characterization of dose-related differences in cognitive impairment across multiple domains.

Taken together, these observations indicate that current aluminum-induced AD models prioritize sensitivity to hippocampal dysfunction while providing a more limited characterization of other cognitive domains relevant to Alzheimer-like neurodegeneration. More diverse and better standardized behavioral assessment strategies may therefore improve both the multidimensional interpretation of cognitive impairment and the comparability of findings across studies.

From a clinical and translational perspective, AlCl_3_-induced rat models are best regarded as controlled models of Alzheimer-like neurotoxicity rather than direct surrogates of sporadic AD in humans. Their main value lies in reproducing convergent behavioral, biochemical, molecular, and histopathological abnormalities under experimentally controlled conditions. At the same time, direct comparison of administered rat doses with human exposure remains difficult. Moreover, the pharmacokinetics of aluminum vary according to the route of administration, so nominal experimental doses cannot be assumed to produce equivalent systemic or brain exposure across paradigms. Oral paradigms may therefore be more clinically intuitive because they better resemble chronic environmental intake, whereas intraperitoneal models provide tighter dose control but are less representative of typical human exposure. In this context, the translational value of AlCl_3_ models lies less in strict human dose equivalence and more in their ability to reproduce convergent neurotoxic processes associated with Alzheimer-like cognitive decline.

Overall, a major challenge in interpreting the current literature is the marked methodological heterogeneity across studies. Differences in AlCl_3_ dose, route of administration, exposure duration, number and type of behavioral tests and outcome reporting substantially limit direct comparison across studies. Although the present review attempted to address this issue by stratifying the evidence according to major experimental variables, the available literature still does not support straightforward one-to-one comparison of all protocols or the identification of a single optimal induction paradigm. Taken together, the available evidence does not support a standardized experimental protocol for AlCl_3_ dose, exposure duration, or behavioral assessment, and this remains a major obstacle to reproducibility and cross-study comparability. Moreover, because experimental regimens varied substantially across studies, the current literature does not support a clear dose–effect or duration–effect relationship at the level of the overall evidence base.

## 5. Conclusions

This review provides a structured overview of the main methodological characteristics of AlCl_3_-induced rat models used in AD research. The available evidence indicates a predominance of oral administration, low-to-moderate AlCl_3_ doses, and subchronic exposure durations, particularly within the 31–60-day range. Behavioral assessment across studies was dominated by a relatively narrow set of hippocampal-dependent paradigms, especially the MWM and the Y-maze, while broader multidomain cognitive profiling remained limited.

Across the reviewed literature, AlCl_3_ exposure was associated with a multidimensional pattern of impairment that extended beyond spatial memory alone and was broadly accompanied by histopathological abnormalities, most consistently affecting the hippocampus and, in some cases, the cortex, together with convergent biochemical and molecular alterations. At the same time, marked heterogeneity in dosing regimens, exposure duration, route of administration, behavioral batteries, and outcome reporting limited direct comparison across studies and did not support a standardized experimental protocol or a clear overall dose–effect or duration–effect relationship. Greater harmonization of experimental design and broader behavioral assessment will be essential to improve the robustness, reproducibility, and translational relevance of aluminum-induced Alzheimer-like rat models.

## 6. Limitations

This review has several limitations. First, the included studies were highly heterogeneous in terms of AlCl_3_ dose, exposure duration, route of administration, behavioral paradigms, and outcome reporting, which reduced direct cross-study comparability. Although we addressed this variability through structured stratification of studies by route, dose range, exposure duration, and type of behavioral assessment, this heterogeneity still limited the integration of findings into a fully unified methodological framework. In addition, the analysis was intentionally restricted to rat studies using AlCl_3_ as the sole induction agent. Although this approach improved species-level consistency and allowed a more focused comparison of behavioral paradigms and model induction parameters, it may limit the generalizability of the findings to other experimental species. The analysis was also restricted to full-text articles published in English. This criterion was applied to ensure accurate and consistent interpretation of study design, methodology, and reported outcomes across the included literature. However, it may also have introduced language-related selection bias, as potentially relevant studies published in other languages may have been excluded. Some oral exposure protocols could not be standardized in mg/kg body weight and were therefore considered only in the qualitative synthesis. Finally, formal quantitative synthesis was further limited by the fact that, even when the same behavioral paradigm was used, outcome measures were interpreted relative to study-specific control groups and experimental conditions. Therefore, apparently similar endpoints, such as escape latency in the MWM, could not be assumed to represent directly comparable effect magnitudes across studies. This issue, together with the heterogeneity in paradigms, dosing regimens, exposure durations, routes of administration, and reporting formats, reduced the methodological reliability of pooled effect-size comparisons and correlations between dose or exposure duration and cognitive deficit severity.

## Data Availability

The data supporting the findings of this review are contained within the article. Further details can be provided by the corresponding authors upon reasonable request.

## Abbreviations

The following list of abbreviations is used in this review:

AD	Alzheimer’s disease
AlCl_3_	Aluminum chloride
CA1	Cornu Ammonis 1 hippocampal region
PRISMA	Preferred Reporting Items for Systematic Reviews and Meta-Analyses
MWM	Morris water maze
NOR	Novel object recognition
